# The Association between Age-Related Macular Degeneration and the Risk of Mortality

**DOI:** 10.1155/2017/3489603

**Published:** 2017-05-18

**Authors:** Peipei Wang, Jie Wang, Jun Ma, Ge Jin, Xueqiang Guan

**Affiliations:** ^1^Department of Cardiology, The Second Affiliated Hospital and Yuying Children's Hospital, Wenzhou Medical University, Xueyuanxi Road, No. 109, Wenzhou, Zhejiang 325000, China; ^2^Department of Neurology, Wenzhou City Hospital of Traditional Chinese Medicine and Western Medicine Combined, Wenzhou, China

## Abstract

Studies have investigated the association between age-related macular degeneration (AMD) and subsequent risks of mortality, but results have been equivocal. We conducted a comprehensive analysis of prospective cohort studies to assess the association of AMD and the risk of mortality in the general population. We searched PubMed and EMBASE for trials published from 1980 to 2016. We included 11 cohort studies that reported relative risks with 95% confidence intervals for the association of AMD and mortality, involving 57,069 participants. In a random-effects model, the adjusted RR (95% confidence interval) associated with AMD was 1.09 (1.02–1.17) for all-cause mortality. Findings from this research provide support that persons with AMD had a higher subsequent risk of mortality than persons without AMD.

## 1. Introduction

Age-related macular degeneration (AMD) is a progressive blinding disease in adults over 50 years of age [1–3]. It is estimated to affect approximately 8.7% of the worldwide population, and the number is anticipated to reach 288 million by the year 2040 [1, 2]. This results in an annual $4.6 billion direct healthcare cost in the United States [4]. As the aging population increases, AMD is becoming a global public health crisis [1]. AMD has both early and late stages [5]. It is affected by the dysfunction of a specialized cell layer in the back of the eye called the retinal pigmented epithelium [1, 5]. Early AMD is usually not associated with loss of vision [6]. And late AMD is manifested through geographic atrophy or the development of neovascularization [4, 6]. Neovascular or wet AMD leads to AMD-related visual loss [1, 6].

Several studies [7–18] have investigated the association between AMD and subsequent risks of mortality, but results have been equivocal. A previous pooling analysis [19] by Wang et al. that included 20 cohort studies that focused on cardiovascular outcomes and all-cause mortality suggested that AMD is associated with an increased risk of all-cause mortality. However, the review omitted many important papers which were eligible and did not investigate the AMD and mortality association according to study characteristics.

Therefore, we conducted a comprehensive meta-analysis of prospective cohort studies to assess the association of AMD and the risk of mortality in the general population.

## 2. Methods

### 2.1. Search Strategy

We conducted a PubMed and EMBASE search through February 2015 for studies describing the association between AMD and the risk of mortality. To make sure our study was based on up-to-date results, we further updated the literature search of PubMed and EMBASE in November 2016. Only papers published in peer-reviewed journals and in English language were considered. In addition, additional studies were identified through the reference lists of relevant publications and relevant reviews. We used search terms “Age-related macular degeneration”, “AMD”, “retina macula degeneration”, “retinal degeneration”, “mortality” and “death” and so on. No attempt was made to identify unpublished reports.

### 2.2. Study Selection

The investigators (P. Wang and J. Ma) independently assessed article eligibility. Any discrepancies regarding eligibility were resolved by consensus. Studies were eligible for our analysis if (1) the authors reported data from an original, peer-reviewed study (i.e., not review articles, letters, comments, or conference abstracts); (2) the main exposure was AMD; (3) the outcome of interest was all-cause mortality; (4) the study was of a prospective cohort design; and (5) relative risk (RR) with corresponding 95% confidence intervals (CIs) was reported in the article. A study must meet all the five inclusion criteria for inclusion. In the case of multiple publications, we chose the articles with the largest sample or the longest follow-up interval. Studies reporting crude associations without any adjustment were also excluded.

The agreement between the two investigators was 99.3% for the first screen and 100% for the full-text articles.

### 2.3. Data Extraction

The standardized, predefined data was extracted from the studies: last name of the first author, publication year, study location, follow-up years, number of cases and participants, mean baseline age, adjustment covariate, and effect size. If the data was not clear from the studies, we corresponded with the author(s) for the relevant data.

### 2.4. Data Synthesis and Analysis

The RR was used estimating association of AMD and the risk of all-cause mortality, and the odds ratio or hazard ratio was considered equivalent to the RR [20]. Forest plots were used to visually assess the RR and corresponding 95% confidence interval across studies. Homogeneity of RR across studies was tested by the Cochrane *Q* statistic (significance level: *P* < 0.10) and the *I*^2^ statistic (ranges from 0% to 100%) [21]. The RR were pooled using the random-effects DerSimonian and Laird models [22]. The possibility of publication bias was evaluated using the visual inspection of a funnel plot [23]. Moreover, subgroup analyses were conducted to evaluate the influences of the selected study and participant characteristics (including regions, case numbers, and types of AMD and number of prescriptions and follow-up year) on the results.

Analyses were performed with the Review Manager software (version 5.2; the Nordic Cochrane Centre, Copenhagen, Denmark). A two-sided *P* < 0.05 was considered statistically significant.

## 3. Results

### 3.1. Literature Search

**Figure 1:**
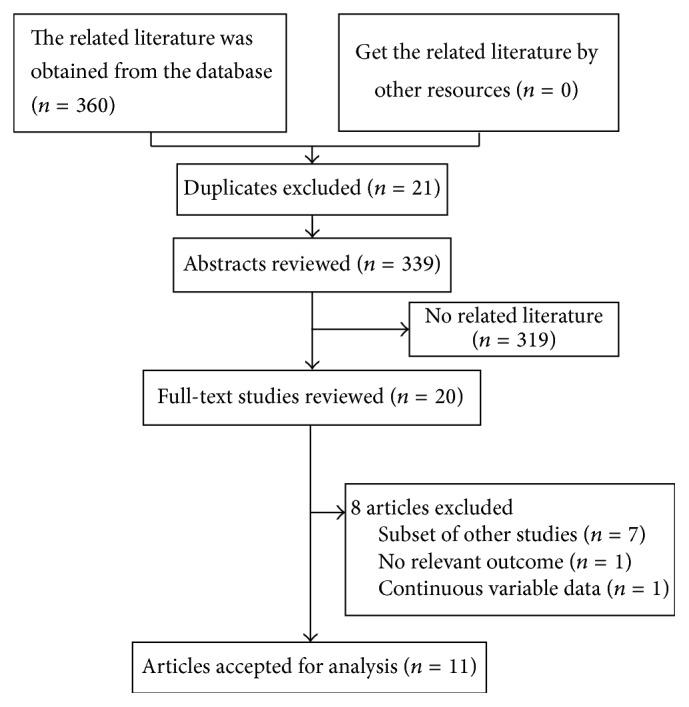
Flow chart of the meta-analysis of AMD and mortality.

Figure 1 shows the literature search flow chart. Our search strategy found 360 articles. After the first round of screening based on titles and abstracts with the aforementioned criteria, 20 articles were selected. Subsequently, after detailed examination, 9 literatures were excluded (reasons shown in Figure 1). No study was retrieved from the reference lists of relevant articles and reviews. As a result, a total of 11 studies were selected for this meta-analysis.

### 3.2. Study Characteristics

The characteristics for the 11 included cohort studies are presented in Table 1. The 11 studies were published between 2001 and 2016. With regard to the study region, two studies were conducted in North America, two in Oceania, three in Asia, and four in Europe. Follow-up duration ranged from 5 to 15 years, with a median of 7.6 years. The sizes of cases ranged from 32 to 1,341, with a sum of 5,213. The sizes of participants ranged from 866 to 13,569 with a sum of 57,069. Most studies included both men and women, and only one study [14] was conducted exclusively in women. AMD ascertainments differed between studies, with most using medical records and some using self-report. Adjustment for potential confounding factors also differed between studies, and most risk estimates were adjusted for age, gender, and body mass index.

### 3.3. Main Analysis

**Figure 2:**
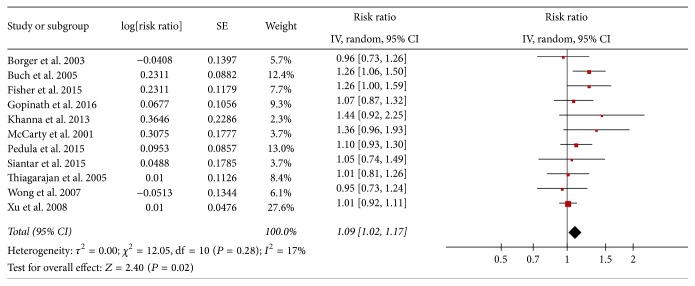
Adjusted relative risks of all-cause mortality associated with AMD.

Among 11 studies, the majority of studies showed positive association (i.e., RR > 1.00) between AMD and the risk of mortality, and only two studies reported RR < 1.00 but not statistically significant. The pooled multivariable-adjusted RR (95% CI) was 1.09 (1.02–1.17; Figure 2), with moderate heterogeneity detected among studies (*I*^2^ = 17%; *P*_heterogeneity_ = 0.28).

**Figure 3:**
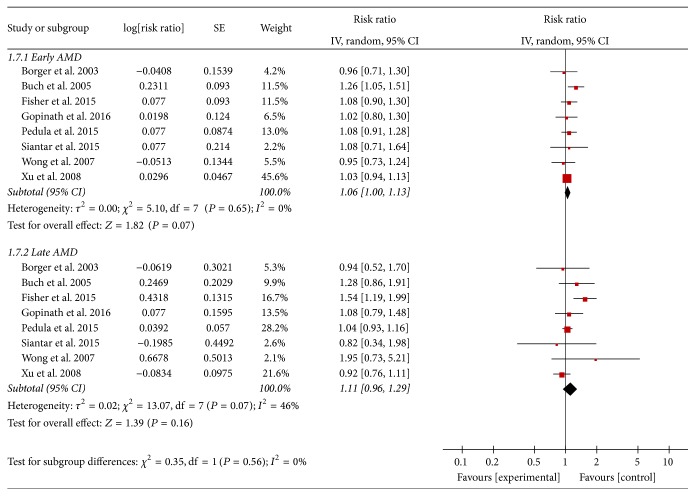
Adjusted relative risks of all-cause mortality associated with different AMD.

We conducted stratified analyses by geographic area, number of AMD, and type of AMD. The results of the stratified analysis are shown in Table 2, and the result of subgroup analysis by types of AMD is presented in Figure 3.

### 3.4. Publication Bias Assessment

**Figure 4:**
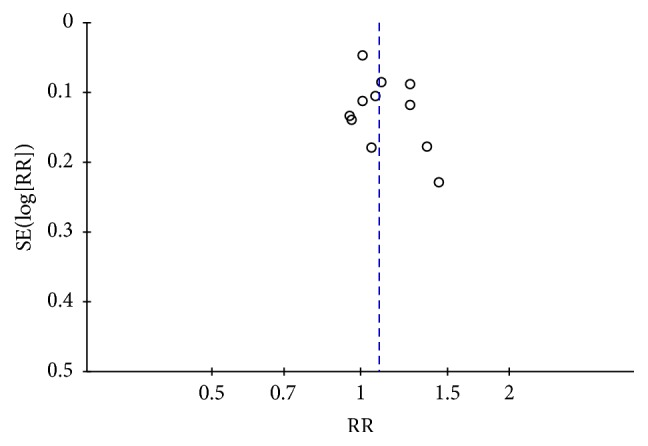
Funnel plots for the detection of public bias among studies that evaluated the associations of AMD with the risk of mortality.

The funnel plot for the detection of public bias among studies that evaluated the associations of AMD with the risk of mortality is shown in Figure 4. The funnel plot was fairly symmetric, indicating that publication bias was not significant.

## 4. Discussion

Our meta-analysis of 11 cohort studies demonstrated significant associations between AMD and all-cause mortality.

For the relationship between AMD and the risk of all-cause mortality, the precise mechanism is not clear. Nevertheless, several possible pathogenic mechanisms have been proposed. AMD could be a marker of underlying serious somatic factors or diseases and reflect the status of systemic processes associated with biological aging, which could be associated with decreased survival and increased biological aging [24]. AMD is a chronic disease of the central retina and is a leading cause of low vision among older adults [2]. Low vision reflects functional status and leads to functional problems, such as accidents, falls, fractures, loss of independence, and depression, all of which may be life-threatening [24–26].

The result was consistent with a previous meta-analysis [19] conducted by Wang et al. suggesting a significant association between AMD and all-cause mortality. However, this review focused on cardiovascular outcomes and cardiovascular disease mortality, and many important papers which were eligible were omitted. What is more, Wang et al. did not investigate the association of AMD and mortality according to study characteristics such as types of AMD. We found a significant association between early AMD and risk of all-cause mortality incidence.

There are several strengths in our study. A major advantage of the meta-analysis is that the present findings are based on cohort studies. Thus, this minimizes the possibility of recall and selection biases. Compared with the previous meta-analysis, the risk estimates reported in the present study were a bit bigger. However, with accumulating evidence and enlarged sample size, we have enhanced statistical power to provide more precise and reliable risk estimates relating between AMD and all-cause mortality.

Limitations also of this meta-analysis should be acknowledged. First, the methods of AMD assessment varied across studies: four studies by ICD code and four studies by self-report from study participants. This may lead to misclassification error and bias. Second, the meta-analysis was limited to English publications, and the possibility of unpublished reports was not yet identified. Third, residual confounding is still possible given that many studies did not adjust for several important potential factors in their models such as physical activity, smoking, unhealthy lifestyle factors, depression, and stress which are also risk factors for poor health outcomes in AMD patients.

## 5. Conclusions

In conclusion, this meta-analysis provides compelling evidence that persons with AMD had a higher risk of mortality than persons without AMD.

## Figures and Tables

**Table 1:** Characteristics of 12 cohort studies of AMD and risk of mortality included in this meta-analysis.

Study: first author, year	Country cohort details	Participants, age (years)	AMD	Mean follow-up, years	Adjusted RR (95% CI)	Adjustment for covariates
Borger et al. 2003	The Rotterdam Study (Netherlands)	6,339, 68.4 (8.6)	581	7	0.96 (0.73, 1.25)	Age, sex, smoking, BMI, cholesterol level, atherosclerosis, hypertension, history of cardiovascular disease

Buch et al. 2005	The Copenhagen City Eye Study (Denmark)	866, 65.5 (5.6)	228	14	1.26 (1.06, 1.51)	Age, sex, smoking status, alcohol consumption, BMI, total cholesterol level, hypertension, cardiovascular disease, diabetes mellitus, any cataract, visual loss (20/40)

Gopinath et al. 2016	BMES (Australian)	3,659 66.2 (9.7)	227	15	1.07 (0.87, 1.32)	Age, sex, qualifications, body mass index, smoking status, alcohol consumption, poor self-rated health, walking disability, presence of hypertension and/or diabetes, doctor-diagnosed history of cancer, angina, stroke and/or acute myocardial infarction

Fisher et al. 2015	AGES (Island)	4,910, 77.0 (5.9)	1341	8.6	1.26 (1.00, 1.59)	Age, gender

Khanna et al. 2013	APEDS (India)	4,188 > 30	32	11	1.44 (0.92, 2.26)	Age, gender, education level, diabetes, hypertension, BMI, smoking status

Pedula et al. 2015	SOF (USA)	1,202, 79.5	487	9.5	1.10 (0.93, 1.30)	Age, race, self-reported frailty, BMI, Mini-Mental State Examination score, walking speed, history of congestive heart failure, history of myocardial infarction, history of chronic obstructive pulmonary disease, history of thiazide diuretic

Siantar et al. 2015	The Singapore Malay Eye Study (Malaysia)	3,273, 58.6 (11.1)	183	7.24	1.05 (0.74, 1.46)	Age, gender, socioeconomic status, hypertension, smoking, BMI, cardiovascular disease

Thiagarajan et al. 2005	United Kingdom	13,569, 81.1 (4.6)	479	6.1	1.01 (0.81, 1.25)	Age, gender, BMI, inability to carry out activities of daily living, presence of a major illness at baseline, history of cardiovascular disease, diabetes mellitus, hypertension, geriatric depression score, daily urinary incontinence, Mini-Mental State Examination score, reported number of falls in the previous 6 months, hearing problems, socioeconomic indicators, self-reported health, low self-reported physical activity levels, smoking history, alcohol intake, social isolation

McCarty et al. 2001	VIP (Australia)	3271, 59	501	5	1.36 (0.96, 1.94)	Univariate analysis

Wong et al. 2007	ARIC (USA)	11,414, range, 49–73	555	8	0.95 (0.73, 1.31)	Age, gender, race, center, education, body mass index, systolic and diastolic BP, diabetes status, total plasma cholesterol and HDL cholesterol, triglyceride, glucose, pack-years of cigarette smoking, current alcohol consumption

Xu et al. 2008	The Beijing Eye Study (China)	4378, 56.1 (10.5)	122	5	1.01 (0.92, 1.10)	Age

BMES: The Blue Mountains Eye Study; AGES: The Age, Gene/Environment Susceptibility Reykjavik Study; SOF: The Study of Osteoporotic Fractures; APEDS: The Andhra Pradesh Eye Diseases Study; BDES: The Beaver Dam Eye Study; VIP: Visual Impairment Project; BMI: body mass index.

**Table 2:** Stratified analyses of mortality associated with AMD.

Group	Number of studies	RR (95% CI)	*P* (heterogeneity)	*I* ^2^ (%)
Total	11	1.09 [1.02, 1.17]	0.28	17
Geographic area				
Oceania	2	1.16 [0.93, 1.14]	0.25	26
North America	2	1.05 [0.92, 1.21]	0.36	0
Asia	3	1.04 [0.92, 1.19]	0.31	14
Europe	4	1.13 [0.99, 1.30]	0.20	36
Number of AMD				
<500	7	1.08 [1.00, 1.16]	0.32	14
≥500	4	1.11 [0.93, 1.32]	0.18	39
Type				
Early AMD	8	1.06 [1.00, 1.13]	0.65	0
Late AMD	8	1.11 [0.96, 1.29]	0.07	46
